# Preconditioning of Mesenchymal Stem Cells with Non-Toxic Concentration of Hydrogen Peroxide Against Oxidative Stress Induced Cell Death: The Role of Hypoxia-Inducible Factor-1

**DOI:** 10.15171/apb.2019.010

**Published:** 2019-02-21

**Authors:** Fatemeh Nouri, Seyed Noureddin Nematollahi-Mahani, Ali Mohammad Shariﬁ

**Affiliations:** ^1^Neuroscience Research Center, Institute of Neuropharmacology, Kerman University of Medical Sciences, Kerman, Iran.; ^2^Department of Anatomy, Afzalipour School of Medicine, Kerman University of Medical Sciences, Kerman, Iran.; ^3^Razi Drug Research Center, Department of Pharmacology, School of Medicine, Iran University of Medical Science, Tehran, Iran.

**Keywords:** Wharton’s jelly derived mesenchymal stem cells, Preconditioning, Hydrogen peroxide, Serum deprivation, HIF-1α, Oxidative stress

## Abstract

***Purpose:*** To investigate the protective effect of preconditioning with non-toxic dose of hydrogen peroxide (H_2_O_2_) as a possible cell signaling molecule, against cell death induced by toxic concentration of H_2_O_2_ or by serum deprivation in human Wharton’s jelly-derived mesenchymal stem cells (HWJ-MSCs) and underlying mechanisms.

***Methods:*** HWJ-MSCs were isolated and identified using flow cytometry. After finding non-toxic concentration of H_2_O_2_, cells preconditioning was performed by H_2_O_2_ (20 μM) for 12 h and cell tolerance against serum deprivation or toxic levels of H_2_O_2_ was assayed by MTT test. Effect of preconditioning on mRNA and protein expression of Akt-1, Bcl-2 and Bax were examined using reverse transcription polymerase chain reaction (RT-PCR) and western blotting respectively. Role of hypoxia-inducible factor (HIF)-1α was explored in presence HIF-1α inhibitor.

***Results:*** Preconditioning with 20 μM H_2_O_2_ for 12 h was non-toxic and decreased cell death induced by oxidative stress and serum deprivation in MSC cultures. However, the increased tolerance reversed in the presence of inhibitor of HIF-1α. By regards to RT-PCR and western blotting data, although expression of Akt-1, Bcl-2 and Bax was not change considerably but phosphorylated Akt-1 (pAkt-1) was up regulated after treatment with 20 μM H_2_O_2_ compared to control group. Moreover after exposure to 100 μM H_2_O_2_, western blotting analysis showed that cell pretreatment with 20 μM H_2_O_2_, decremented Bax/Bcl2 ratio and up-regulated HIF-1α and pAkt-1 compared to the control group.

***Conclusion:*** Increased tolerance of H_2_O_2_-pretreated cells led to the suggestion that transplantation of H_2_O_2_ preconditioned MSCs may improve therapeutic potential of stem cells in cell therapy procedures.

## Introduction

Stroke as a destructive disease and one of the world’s biggest causes of death has led to more than 5 million deaths in 2016.^1^ despite wide investigations on the mechanisms of stroke, efficient therapies to restore lost neurological function after stroke, has not been achieved yet. Several studies have underlined the potential of stem cell transplantation as a novel therapeutic strategy for stroke recovery. Stem cells may migrate into damaged regions of brain and differentiate into neural cells or may promote endogenous neurogenesis and angiogenesis through the secretion of trophic factors in the injured regions of central nervous system.^2-4^

High degree of transplanted cell mortality is a limiting factor for the successful clinical cell therapy.^5,6^ Therefore, development of strategies that lead to increase survival of transplanted cells may improve therapeutic potential of stem cell-based therapies.^7^ Earlier investigations indicated that ischemic preconditioning can obviously increase the resistance of treated cells or tissues following stricter conditions.^8^

Ischemic preconditioning stimulates phosphatidylinositol 3-kinase** (**PI3K)/Akt signaling pathway which enhances Akt-1 phosphorylation and up-regulation of anti-apoptotic Bcl-2 protein.^9-11^ Bcl-2 as an important anti-apoptotic protein, is localized in the membrane of mitochondria and stabilizes mitochondrial membrane permeability. Bax, another protein of Bcl-2 family, induces apoptosis as it binds to Bcl-2 and antagonizes its anti-apoptotic effect.^12^ an increase in the ratio of Bax to Bcl-2 has been demonstrated as a predictor for apoptosis progression. The increase in the ratio of Bax to Bcl-2 is associated with the increased of mitochondrial membrane permeability and mitochondrial cytochrome c release.^13,14^

Protective properties under ischemic preconditioning is in part mediated by hypoxia-inducible factor-1 (HIF-1), as an important transcription factor. HIF-1 is a heterodimeric transcription factor that is consisted of two subunits, HIF-1α and HIF-1β. HIF-1α is known as an unstable protein, is hydroxylated by prolyl-4 hydroxylase domain family (PHD) proteins and afterwards, it is ubiquitinated and tagged for degradation by the proteasome. Since, oxygen and iron (II) are essential for activity of PHD, thus in hypoxic conditions as well as in the presence of iron chelators, degradation of HIF-1α is not occurred and it is dimerised with the HIF-1β subunit to form active transcription factor, HIF-1, which affects target genes, that contribute to the cell survival and apoptosis.^15,16^

Ischemic preconditioning is considered to promote the production of reactive oxygen species (ROS) at signaling levels. The role of ROS is paradoxical; while at high levels it can contribute in damaging cellular compounds and initiating cell death, but at low levels, ROS act as cell signaling molecules and may play an essential role in protection against the destructive conditions.^17,18^ However hydroxylation of HIF-1α by PHD enzymes is under the control of ROS. ROS contribute to inactivation of PHD, subsequently HIF1-α would escape from degradation and participate in HIF-1 formation.^19^

Although the molecular mechanisms involved in ischemic preconditioning has been extensively studied, the role of HIF-1 and apoptotic pathways require more attention. The present article is intended to focus on the preconditioning with non-toxic levels of H_2_O_2_ to explore the role of ROS in ischemic preconditioning.

The presented study has investigated the preconditioning effect of H_2_O_2_ (as an important signaling molecule in ischemic preconditioning) on the resistance of human Wharton’s jelly-derived mesenchymal stem cells (HWJ-MSCs) under high concentration of H_2_O_2_ treatment and serum deprivation (as important causes of cell damage and cell death during ischemic stroke), and the mechanism involved with HIF-1α.

## Materials and Methods

### HWJ-MSC isolation

Human umbilical cord was obtained from full term deliveries which had no complications throughout pregnancy. According to the formerly described procedures,^20^ Wharton’s jelly was cut into 2-5 mm pieces and seeded in a cell culture dish with DMEM-F12 containing 10% Gibco fetal bovine serum (FBS), penicillin (100 units/mL), streptomycin (100 µg/mL) and 2.5 μg/mL amphotericin B (2.5 μg/mL) at 37°C to allow cells to migrate from the margins of the explants. Fifty percent of the culture media was refreshed every 5 days and the cells reached approximately 80% during 2 weeks confluence. At this time, the cells were detached and sub-cultured to a new flask for further use.

### Flow cytometry

The surface marker expression of isolated HWJ-MSCs was investigated using flow-cytometry according to the described procedure.^20^ In brief, 1×10^5^ cells/mL (passage three) were washed with PBS, fixed and permeabilized with 0.2% Triton X-100 and incubated with normal goat serum diluted in PBS (1:9) for 15 min at 4°C for blocking non-specific binding of the primary antibodies. The cells were incubated with the specific antibody conjugated with fluorescein isothiocyanate (FITC) or phycoerythrin (Santa Cruz) at concentrations recommended by the respective manufacturer’s for 1 h and then analyzed by a flow cytometer. The antibodies conjugated by FITC were: anti-CD44, anti-CD34 (Chemicon, USA) and anti-CD45 (eBioscience, USA), and those conjugated by PE were anti-CD73 (Becton Dickinson [BD], USA), anti- CD90 (Dako, Denmark) and anti-CD105 (R&D, USA). Following incubation, the cells were washed with 2% FBS in PBS and run through a BD FACSCalibur (USA) flow cytometer. The control group was stained with isotype-matched antibodies (FITC- and PE-conjugated mouse IgG monoclonal isotype standards), which were confirmed by positive fluorescence of the limbal samples. Typically for each tube, 10 000 events were collected and the data analysis was performed using WinMDI software (BD Biosciences, USA).

### Adipogenic and osteogenic differentiation

To verify the multipotency of HWJ-MSCs derived from the culture media, the cells were differentiated into the adipogenic and osteogenic lineages according to the formerly described procedure.^20,21^ Briefly, HWJ-MSCs at fourth passage were seeded at the density of 50 000 cells/well in a 6-well plate for 48 h. The culture medium was then changed to adipogenic induction medium contained DMEM-F12, 10% FBS, 1% (v/v) penicillin-streptomycin, ascorbate-phosphate (50 µg/mL), indomethacin (50 µg/mL), and dexamethasone (100 nM) for 3 weeks. The same procedure was done for osteogenic induction except that osteogenic medium containing ascorbate-phosphate (50 µg/mL), β-glycerophosphate (10 mM) and dexamethasone (10 nM) was added for 3 weeks. The culture media were refreshed every 3 days. Control cells were cultured in DMEM-F12 supplemented with 10% FBS and antibiotics. For adipogenic and osteogenic differentiation, the induced cells were stained with Oil Red O and Alizarin Red-S staining solutions respectively. The cells were visualized under a light microscope (Nikon S100, Japan).

### Assessment of HWJ- MSCs viability following treatment with different concentration of H_2_O_2_

To find the optimum concentration of H_2_O_2_ on HWJ-MSCs viability, the cells were treated with different concentrations (0, 10 µM, 20 µM, 50 µM and 100 µM) of H_2_O_2_ for 12 h. Percentage of viable cells was measured by the methyl thiazol tetrazolium bromide (MTT) assay. This colorimetric assay is based on cleavage of tetrazolium salts to form an insoluble purple formazan product in the metabolically activated cells that can be quantified using a spectrophotometer. In brief, HWJ-MSCs were cultured at a density of 5000 cells/well in a 96-well plate and treated with different concentrations of H_2_O_2_ for 12 h. After incubation, the cells were treated with MTT solution (0.5 mg/mL) at 37°C for 2 h. Afterwards, the culture medium was removed, and the purple formazan crystals were dissolved in 100 μL dimethylsulfoxide (DMSO). The absorbance of each well was measured at 570 nm using ELISA reader (Start Fax-2100, UK) with reference wave length of 630 nm.

### Serum Deprivation on preconditioned HWJ-MSCs with H_2_O_2_

To investigate the tolerance of the cells preconditioned with H_2_O_2_ against serum deprivation (SD), as a suitable in vitro model for studying the ischemic regions in stroke, the HWJ-MSCs at a density of 5000 cells/well were seeded in a 96-well plate. After 24 h, the cells were treated with 20 μM H_2_O_2_ for 12 h. The wells were rinsed with serum-free medium 3 to 5 times and were then incubated with DMEM containing 1% (v/v) penicillin-streptomycin without FBS for 24 h under the conditions described for cell culture. To investigate the role of HIF-1α on the regulation of cell tolerance against serum deprivation, some cultures were treated with 30 µM HIF-1α inhibitor (sc-205346) for 1 h and then 20 µM H_2_O_2_ was added for 12 h. The cells were then washed with basal medium and incubated with FBS free media containing DMEM-F12 and 1% penicillin-streptomycin for 24 h. The viability of cells was measured with MTT test and compared with the control groups.

### Damaging treatment by H_2_O_2_ on preconditioned HWJ-MSCs

To investigate the tolerance of cells preconditioned with non-toxic concentration of H_2_O_2_ against H_2_O_2_ induced cell death, as a common lethal insult in the ischemic regions of cerebral stroke, after 12 h preconditioning of HWJ-MSCs with 20 μM H_2_O_2_, the cells were washed with basal medium three times. Afterwards, the cells were incubated with 100 μM H_2_O_2_ in DMEM-F12 containing 10% FBS, 1% (v/v) penicillin-streptomycin for 24 h. The viability of cells viability was determined using MTT assay. To study the role of HIF-1α on regulation of cell tolerance against H_2_O_2_, some cultures were treated with 30 µM HIF-1α inhibitor for 1 h and then 20 µM H_2_O_2_ was added to the cells for 12 h. The cells were then washed with basal medium and incubated with 100 µM H_2_O_2_ in DMEM-F12, 10% FBS and 1% penicillin-streptomycin for 24 h and cell viability was then measured by MTT test.

### Semi-quantitative RT-PCR

To investigate the relative fold change of genes expressions (Bax, AKT-1, Bcl-2) involved in the survival of the cells pretreated with non-toxic level of H_2_O_2_ (20 μM for 12 h), semi-quantitative RT-PCR (sqRT-PCR) was performed. To investigate the molecular mechanism of HIF-1α on the expression of AKT-1, Bcl-2, and Bax after 1 h pretreatment with 30 µM HIF-1α inhibitor, 20 μM H_2_O_2_ was added to the cells for 12 h. Total RNA was isolated by TRIZOL reagent (Invitrogen) according to the manufacturer’s instructions and was quantified spectrophotometrically. First-strand cDNA was synthesized from 1 μg RNA in the presence of 2 μg/μL oligo (dT) primer (Fermentas) and 200 U MMLV (Fermentas) in a total volume of 20 μL. The reaction mixture was respectively incubated at 42°C for 1 h and at 72°C for 10 min. Aliquots (5 μL) of cDNA was subjected to PCR using specific primers (Table 1).

**Table 1:** Primers used for semi-quantitative RT-PCR

**Gene**	**Primer Sequence (5‘–3‘)**
Akt-1	F:ATTGTTCTGAGGGCTGAGGC
	R:TGGACGATAGCTTGGAGGGA
Bcl-2	F:TTAGCCCCCGTGACCTCTTA-3ˊ
	R:TGTGCTGCTATCCTGCCAAA
Bax	F:GCCTCACTCACCATCTGGAA
	R:TTACCCCCTCAAGACCACTCT
β-Actin	F:CACCATGGATGATGATATCGC
	R:AGTCCATCACGATGCCAGTG

The PCR conditions were as follows: hot start at 95°C for 10 min; 35 cycles of 94°C for 30s, 53°C for 1 min and 72°C for 3 min followed by a final extension at 72°C for 5 min. PCR was performed for β-actin as the internal control. PCR products were separated on a 1.5% agarose gel and visualized by Nancy-520 stained agarose gel electrophoresis. To study the cytoprotective effect of initial pretreatment with H_2_O_2_ by challenge with cytotoxic levels of H_2_O_2_, the expression of these genes was measured using sqRT-PCR after 12 h pretreatment with 20 μM H_2_O_2_ followed by the treatment with 100 µM H_2_O_2_ for 24 h.

To investigate the molecular mechanism of HIF-1α on this tolerance, the expression of these genes was measured using sqRT-PCR after 1 h pretreatment with 30 µM HIF-1α inhibitor, next adding 20 μM H_2_O_2_ for 12 h, then rinsing with basal medium and subsequently treatment with 100 μM H_2_O_2_ for 24 h and compared with cultured cells pretreated with 20 μM H_2_O_2_ for 12 h and then treated with 100 μM H_2_O_2_ for 24 h as positive control. Moreover, the gene expression of this group compared with treated culture just with 100 μM H_2_O_2_ for 24 h without pretreatment with non-toxic level of H_2_O_2_ as negative control.

### Western blot analysis

To analyze the difference between the levels of selected protein contributed to cell survival (HIF-1α, Bcl-2, Bax, Akt-1 and pAkt-1), in the cells pretreated with non-toxic concentration of H_2_O_2_, western blotting analysis was performed. The cells were exposed to 20 μM H_2_O_2_ for 12 h. The non-treated and treated cells with H_2_O_2_ were examined with western blotting. To investigation of the role of HIF-1α some groups were pretreated with 30 µM HIF-1α inhibitor before adding 20 μM H_2_O_2_ and were examined with western blotting. The cells were dissociated using trypsin and washed two times with ice-cold PBS. Then, cells were lysed in 0.2 mL of RIPA lysis buffer (10 mM Tris-HCl, pH 7.4, 5 mM EDTA, 150 mM NaCl, 0.1% sodium dodecyl sulfate (SDS), 1% Triton X-100 and 0.5% sodium deoxycholate) containing protease and phosphatase inhibitor cocktails (Sigma-Aldrich) and centrifuged at speed of 15 000 g for 20 min at 4°C. The concentration of protein was measured using Bradford Protein Assay. Equal amounts of proteins from each sample were loaded and separated by sodium dodecyl sulfate polyacrylamide gel electrophoresis (SDS-PAGE) and transferred onto a polyvinylidene difluoride (PVDF) membrane (Invitrogen). Blots were then blocked with 2% skim milk for 1 h at room temperature. After blocking, the blots were incubated with antibodies including HIF-1α (ab82832; 1:500), Bcl-2 (ab6201; 1:100), Bax (ab7977; 1:1000), pan-AKT-1 (ab8805; 1:500) and phospho AKT-1 (ab66138; 1:5000) overnight at 4°C. The membrane was washed twice with 0.1% Tween 20, TBST, and 2% skim milk for 10 min. Then, they were incubated with anti-rabbit antibody (Cell Signaling Technology) conjugated with horseradish peroxidase (HRP) or incubated with anti-mouse antibody (Cell Signaling Technology) for 1 h at room temperature according to the experiment design. Finally, the protein bands were detected by enhanced chemiluminescence (ECL) reagent (Pierce ECL Western Blotting Substrate, Amersham Biosciences). In the loading control proteins, the membranes were probed with a rabbit anti-β-actin antibody (Cell Signaling Technology). Protein bands were analyzed by an analysis software (TotalLab software, Wales, UK). To study the cytoprotective effect of pretreatment with non-toxic concentration of H_2_O_2_ against H_2_O_2_ induced cell death, the expression of these proteins was measured using Western blotting after 12 h pretreatment with 20 µM H_2_O_2_ followed by the treatment with 100 µM H_2_O_2_ for 24 h and compared with those that just treated with 100 µM H_2_O_2_ without pretreatment with non-toxic concentration of H_2_O_2_. To explore the protective effect of H_2_O_2_ on cell survival mediated by HIF-1α, the expression of these proteins was measured using Western blotting after 1 h pretreatment with HIF-1α inhibitor, next adding 20 μM H_2_O_2_ for 12 h and then, rinsing with basal medium and subsequently treatment with 100 μM H_2_O_2_ for 24 h. The cells that were pretreated with 20 μM H_2_O_2_ for 12 h and then with 100 μM H_2_O_2_ for 24 h, were regarded as positive control while the cells that just treated with 100 μM H_2_O_2_ for 24 h, were regarded as negative control.

## Results and Discussion

### HWJ-MSC isolation and in vitro culture

**Figure 1:**
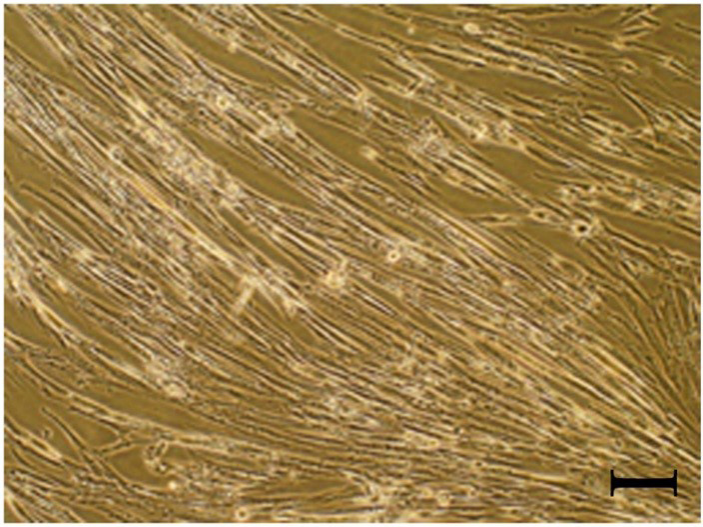
Morphology of HWJ-MSC. Cells showed fibroblast or spindle-like feature. Parallel or whirlpool like arrangement of bipolar spindle like cells with high confluency (> 90%). Scale bar 50 µm.

Cells morphology was visualized by the inverted light microscope (Nikon S100, Japan). 10 days after explanation, the cells migrated from fragments were detectable while they were heterogeneous in appearance. After 3 passages, human WJ-MSCs became relatively homogeneous and elongated (fibroblast-like or spindle-shaped) (Figure 1).

### Flow cytometry

To analyze MSCs (CD44, CD105, CD90 and CD73) and hematopoietic (CD34 and CD45) markers, flow cytometry was used. The analysis showed that MSCs markers were positive while hematopoietic markers were negative.

### Adipogenic and osteogenic differentiation

In adipogenic differentiation, lipid droplets appeared as cherry red spheres within individual cells by Oil Red O staining solution. Similarly, the ground matrix stained positively with Alizarin-Red staining solution at the end of the third week following the osteogenic induction. Control group (non-treated cells) showed no adipocyte-like or osteoblast-like transformation even after 3 weeks of cultivation.

### Effect of H_2_O_2_ on viability of HWJ-MSCs

**Figure 2:**
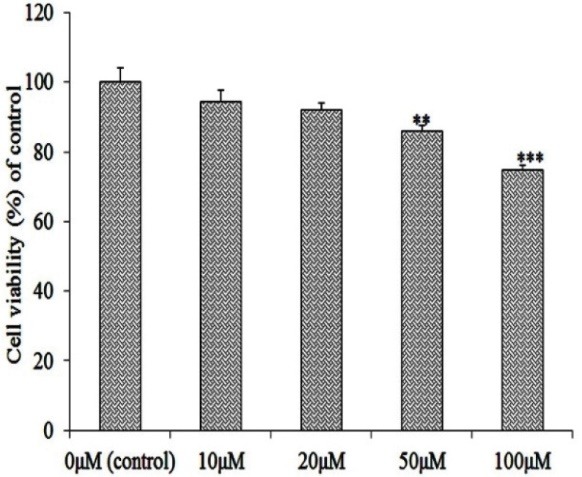
Total cell viability after treatment with H_2_O_2_. The cells were treated with different concentrations of H_2_O_2_ for 12 h. Viability of total cells was measured by MTT test. Groups that treated with 0 µM H_2_O_2_ has regarded as control group in all tests, results are reported as the mean ±SEM (n=3) and are significant at ** *P*˂0.01 and *** *P*˂0.001 as compared to control.

**Figure 3:**
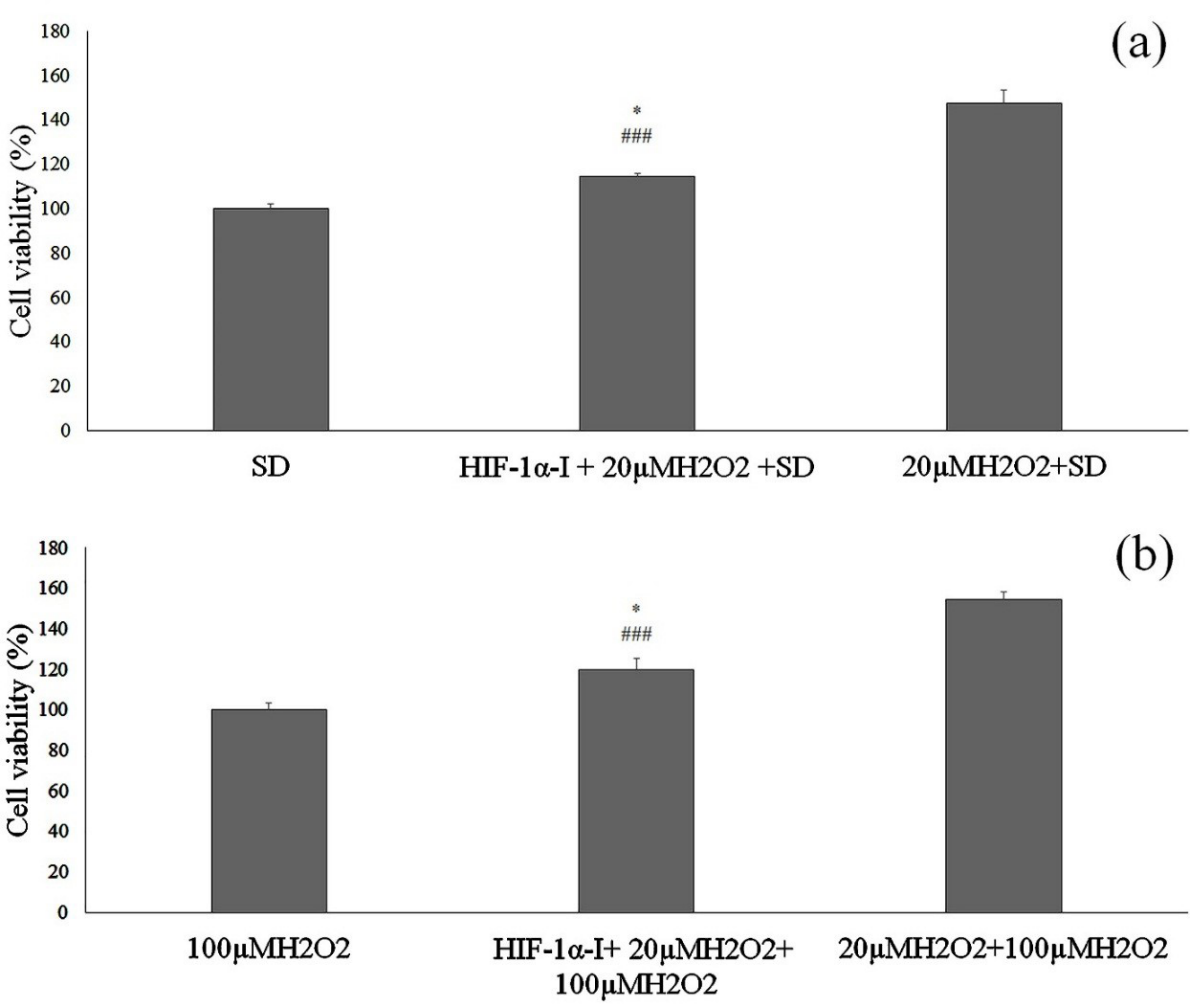
Effect of HIF-1α inhibitor (HIF-1α-I) on MSCs viability after serum deprivation (SD) or exposure to 100 µM H_2_O_2_. (a) The cells treated with 20 µM H_2_O_2_ and serum withdrawal have regarded as positive control group while the cells treated with serum-deprived media (without pretreatment with 20 µM H_2_O_2_) has regarded as negative control group. (b) The cells treated with 20 µM H_2_O_2_ and 100 µM H_2_O_2,_ were regarded as a positive control group and the cells treated with 100 µM H_2_O_2_ were regarded as negative control group. Results were reported as the mean ± SEM (n=3) and were significant at * *P<*0.05 as compared to negative control group and at ^###^*P<*0.001 as compared to positive control group.

To find the optimum concentration for preconditioning with H_2_O_2_ for 12 h on HWJ-MSCs, viability was evaluated using MTT assay. Results showed that H_2_O_2_ could decrease total cell viability at the concentration manner compared with the control group. Nonetheless, the cytotoxicity at the concentrations of 10 µM and 20 µM after 12 h, was not significant. We selected 20 µM H_2_O_2_ at time point of 12 h for the rest of study. Increasing the concentration, 100 µM H_2_O_2_ significantly reduced the cell viability. The tolerance of HWJ-MSCs to damage was assessed 24 h later by challenge with 100 µM H_2_O_2_ (Figure 2). Effect of preconditioning with 20 µM H_2_O_2_ on cell death induced by Serum Deprivation MTT assay data disclosed that cells preconditioned with media rich in 20 µM H_2_O_2_ and poor of FBS and HIF-1α inhibitor had the highest cell viability whereas the non- preconditioned cells with H_2_O_2_ and just treated with serum free media showed the lowest viability. Cell viability of the pretreated culture with HIF-1α inhibitor and 20 µM H_2_O_2_, was significantly lower than non-pretreated culture with HIF-1α inhibitor and was higher than non-pretreated cells with 20 µM H_2_O_2_, as well (Figure 3).

### Effect of preconditioning with 20 µM H_2_O_2_ on cell death induced by high H_2_O_2_ or by serum deprivation

To investigate the tolerance of preconditioned cells with non-toxic concentration of H_2_O_2_ against H_2_O_2_ -induced cell death, the cells were treated with 100 µM H_2_O_2_ for 24 h with or without preconditioning with 20 µM H_2_O_2_ for 12 h. Also in order to study the mechanism mediated by HIF-1α, a group was pretreated with inhibitor of HIF-1α for 1 h and then with 20 µM H_2_O_2_ for 12 h. These cells were exposed to 100 µM H_2_O_2_ for 24 h and their viability was assayed by MTT test. The results demonstrated that the cell viability in the cells treated with 100 µM H_2_O_2_ without preconditioning with non-toxic level of H_2_O_2_ was significantly decreased compared to those preconditioned with initial concentration of H_2_O_2_. However, after exposure to 100 µM H_2_O_2_, cell viability of the pretreated cells with HIF-1α inhibitor and non-toxic concentration of H_2_O_2_ was significantly less than non-pretreated cells with HIF-1α inhibitor (Figure 3).

These data are in good agreement with a previous study by Tang et al who disclosed that preconditioning of PC12 cells with 10 µM or 20 µM H_2_O_2_ could significantly protect cells against apoptosis induced by high concentration of H_2_O_2_.^22^

Taban et al in a report consistent with our results demonstrated that co- preconditioning of MSCs with low serum and H_2_O_2_ has prevented cell death induced by 300 µM H_2_O_2_.^23^ McAdle et al reported that the pretreatment of cultured skeletal muscle myotubes with a non-damaging concentration of H_2_O_2_ caused significant protection against damaging treatment of H_2_O_2_.^24^

Furthermore, Chang et al indicated that treatment of primary cortical neurons with non-toxic concentration of H_2_O_2_ induced protection against oxygen glucose deprivation (OGD) compared to control cells.^25^

In an in vivo study, Valen et al showed functional protection of preconditioning with low dose of H_2_O_2_, against cardiac dysfunction induced by high levels of H_2_O_2_ in Langendorff-perfused rat heart model.^26^

### Effect of H_2_O_2_ on gene expression in HWJ-MSCs

**Figure 4:**
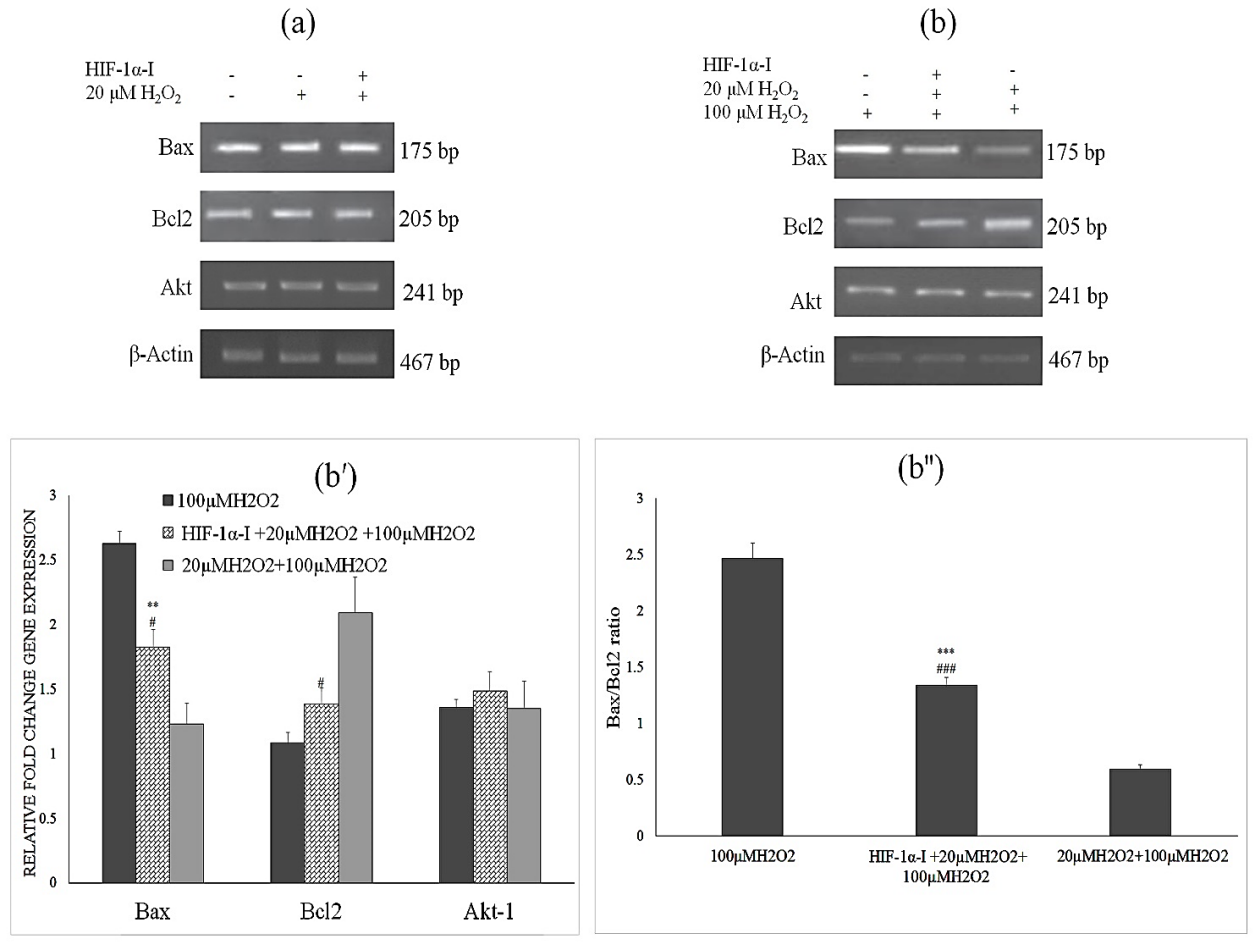
Regulation of gene expression under 20 µM H_2_O_2_ preconditioning. (a) The gene expression pattern of Bax, Bcl-2 and Akt- with pretreatment by HIF-1α inhibitor (HIF-1α-I) for 1h before adding 20 µM H_2_O_2_ for 12h. (b) mRNA levels after the termination of 100 µM H_2_O_2_ exposure. (b′) Quantitative analysis of gene expression was performed by densitometry. β-Actin was used as a loading control. Data were represented as mean ± SEM. ** *P* < 0.01 versus non-pretreated cells with 20 µM H_2_O_2_ before exposure to 100 µM H_2_O_2_ and ^#^*P* < 0.05 versus pretreated cells with 20 µM H_2_O_2_ before exposure to 100 µM H_2_O_2_ (n=3). (b’’) The Bax/Bcl2 ratio. *** *P* < 0.001 versus non-pretreated cells with 20 µM H_2_O_2_ before exposure to 100 µM H_2_O_2_ and ^###^*P* < 0.001 versus pretreated cells with 20 µM H_2_O_2_ before exposure to 100 µM H_2_O_2._

To investigate the relative fold changes in the gene expression of cells treated with non-toxic concentration of H_2_O_2_, sqRT-PCR was performed. SqRT-PCR analysis showed that the changes in the mRNA expression of the Bcl-2 and Bax genes was not significant after the treatment with 20 μM H_2_O_2_ for 12 h as compared to the control group. After exposure to 100 µM H_2_O_2_, sqRT-PCR analysis of the cells without preconditioning with 20 µM H_2_O_2_ showed a significant decrease in Bcl-2, and over- expression of Bax as compared to the cells preconditioned with non-toxic concentration of H_2_O_2_ while, Akt-1 expression did not significantly change. However, after exposure to 100 µM H_2_O_2_, Bax/Bcl2 ratio in the HIF-1α inhibitor pretreated cells was higher than the cells that were preconditioned just with 20 µM H_2_O_2_. This ratio was lower than the cells that were not preconditioned with 20 µM H_2_O_2_ and HIF-1α inhibitor (Figure 4).

### Effect of preconditioning with 20 μM H_2_O_2_ on cell death induced by high H_2_O_2_ or by serum deprivation

**Figure 5:**
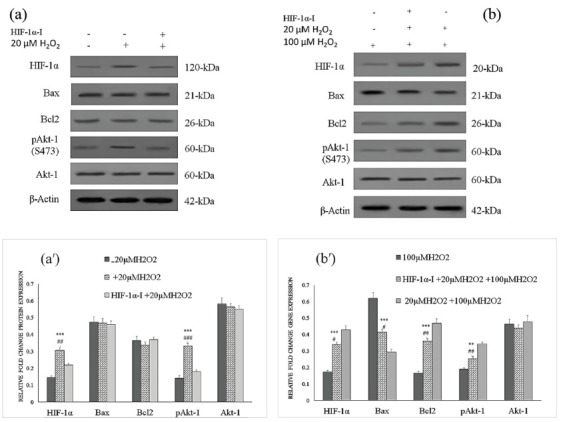
20 µM H_2_O_2_ preconditioning induced protein regulation. (a) The protein levels of HIF-1α, Bax, Bcl-2, pAkt-1and Akt- with pretreatment by HIF-1α inhibitor (HIF-1α-I) for 1 h before adding 20 µM H_2_O_2_ for 12 h. β-actin was used as a loading control. (a′) Quantitative analysis of protein expression was performed by densitometry. ***P < 0.001 versus non-treated cells with 20 µM H_2_O_2_ while ^##^*P* < 0.01 and ^###^*P* < 0.001 versus pretreated cells with HIF-1α-I and 20 µM H_2_O_2_. (b) Protein levels after the termination of 100 µM H_2_O_2_ treatment. (b′) Quantitative analysis of protein expression was performed by densitometry. **P < 0.01 and ***P < 0.001 versus non-pretreated cells with 20 µM H_2_O_2_ before exposure to 100 µM H_2_O_2_ while ^#^*P* < 0.05 and ^##^p˂0.01 versus pretreated cells with 20 µM H_2_O_2_ before treatment with 100 µM H_2_O_2_ (n=3).

To analyze the difference between the selected protein levels in different groups, Western blotting was used. At the protein level, up-regulation of pAkt-1 and HIF-1α after 12 h treatment with 20 μM H_2_O_2_ was observed, while Bax/Bcl2 ratio and total Akt-1 protein expression was not significantly changed as compared to control group. The group that was pretreated with HIF-1α inhibitor and H_2_O_2_, showed a shift in the expression of pAkt-1 and HIF-1α to the control group. In the cells which were treated with 100 µM H_2_O_2_ and without preconditioning with non-toxic concentration of H_2_O_2_, Bax/Bcl2 ratio significantly increased as compared to the cells preconditioned with 20 µM H_2_O_2_. However, the protein expression pattern of the cells pretreated with HIF-1α inhibitor and 20 µM H_2_O_2_ and then exposed to 100 µM H_2_O_2_ displayed a shift to non- preconditioned cells with non-toxic level of H_2_O_2_, as evidenced by increase in Bax/Bcl2 ratio and decrease in HIF-1α and pAkt-1 levels (Figure 5).

Previous studies have shown successful HIF-1α stabilization followed by the treatment with H_2_O_2_.^27,28^

Besides, western blot analysis of pretreated cells with 20 µM H_2_O_2_ showed that after challenging with 100 µM H_2_O_2_, pAkt-1 expression increased while Bax/Bcl2 ratio decreased as compared to the controls. Moreover, inhibition of HIF-1α with HIF-1α inhibitor in cells pretreated with H_2_O_2_ caused decrement in pAkt-1 level and increment in Bax/Bcl2 ratio as compared to non-pretreated cells with HIF-1α inhibitor.

The results of this study were consistent with the previous reports, indicating that ROS can induce Akt-1 phosphorylation in different cell types, such as articular chondrocytes.^29,30^ mammary epithelial cells,^31^ adipocytes,^32^ metanephric mesenchymal cells,^33^ and skeletal muscle precursor cells.^34^

In agreement with the current observations, HIF-1α was involved in activation of PI3K/Akt signaling pathways reported in our previous study.^21^

In good agreement with a previous study by Tang et al, they showed that reduced Bcl-2 expression and increased ROS levels under high concentration of H_2_O_2_, were blocked by preconditioning with non-toxic concentration of H_2_O_2_. Also their study found that preconditioning with 10 µM H_2_O_2_ induced overexpression of Bcl-2.^22^

Moreover, Chang et al indicated that treatment of primary cortical neurons with non-toxic concentration of H_2_O_2_ resulted in higher HIF-1α protein expression.^25^

Since the stem cells seem to be critical players in the future of regenerative medicine, their response to ischemic conditions and sudden change in the cells environment after transplantation have been considered by some researchers.

This study was designed to investigate the protective effects of preconditioning with non-toxic concentrations of H_2_O_2_, as an important cell signaling molecule during ischemic preconditioning, against cytotoxic effects of serum deprivation or toxic levels of hydrogen peroxide on HWJ-MSCs in vitro and the underlying mechanism with HIF-1α. Since, we hypothesized that serum provides sustenance and trophic factors for cells in culture so, cell culture with serum deprivation can be used as a suitable in vitro model for ischemic regions in stroke. In addition, it has been theorized that H_2_O_2_ preconditioning at non-toxic concentration makes cells resistant to death induced by high levels of H_2_O_2_ as a common lethal factor in ischemic regions and in several degenerative disorders.

In summary, for the first time our data disclosed that treatment of Wharton’s jelly MSCs with 20 µM H_2_O_2_ for 12 h enhanced cell tolerance against toxic levels of H_2_O_2_ partly by overexpression of HIF-1α protein. Notably, cell death induced by serum deprivation was dependent on HIF-1α activation. However, to complete this study, further investigations are still required to understand more detailed mechanisms involved in and a parallel study to investigate the effect of H_2_O_2_ on MSCs transplanted in vivo models.

## Conclusion

The present study demonstrated an increase in cell survival of preconditioned cells with non-toxic concentration of H_2_O_2_ after exposure to toxic levels of H_2_O_2_ or after culturing in serum free media partially dependent on HIF-1 activation. Regarding to these results, we suggest that transplantation of preconditioned MSCs with low concentration of H_2_O_2_ may improve tolerance and therapeutic potential of stem cells within damaged tissues in in-vivo studies and may represent a clinically appropriate approach in cell therapies.

## Ethical Issues

All the procedures were approved by the Ethics Committee of Kerman University of Medical Sciences and a consent form was obtained from parents after caesarean section.

## Conflict of Interest

The authors confirm that this article has no conflict of interest.

## Acknowledgments

The authors especially thank S. Tavakol from Cellular and Molecular Research Center, Iran University of Medical Sciences, Tehran, Iran.
